# Neighborhood Safety and Adipose Tissue Distribution in African Americans: The Jackson Heart Study

**DOI:** 10.1371/journal.pone.0105251

**Published:** 2014-08-28

**Authors:** Do Quyen Pham, Mark J. Ommerborn, DeMarc A. Hickson, Herman A. Taylor, Cheryl R. Clark

**Affiliations:** 1 Center for Community Health and Health Equity, Brigham and Women’s Hospital, Boston, Massachusetts, United States of America; 2 Department of Medicine, University of Mississippi Medical Center, Jackson, Mississippi, United States of America; German Diabetes Center, Leibniz Center for Diabetes Research at Heinrich Heine University Duesseldorf, Germany

## Abstract

**Objective:**

Patterns of fat distribution are heavily influenced by psychological stress, sex, and among women, by menopause status. Emerging evidence suggests the lack of perceived neighborhood safety due to crime may contribute to psychological stress and obesity among exposed residents. Our objective is to determine if perceived neighborhood safety is associated with abdominal adiposity among African-American men and women, and among pre- and postmenopausal women in the Jackson Heart Study.

**Design and Methods:**

We examined associations between perceived neighborhood safety, fat distribution, and other individual-level covariates among Jackson Heart Study participants (N = 2,881). Abdominal adiposity was measured via computed tomography scans measuring the volumes of visceral, subcutaneous and total adipose tissue. We also measured body mass index (BMI), and waist circumference. Multivariable regression models estimated associations between perceived neighborhood safety, adiposity, and covariates by sex and menopause status.

**Results:**

Adjusting for all covariates, women who strongly disagreed their neighborhood was safe from crime had a higher BMI compared to women who felt safe [Std B 0.083 95% CI (0.010, 0.156)]. Premenopausal women who felt most unsafe had higher BMI, waist circumference, and volumes of visceral and total adipose tissue than those who felt safe [Std B 0.160 (0.021, 0.299), Std B 0.142 (0.003, 0.280), Std B 0.150 (0.014, 0.285), Std B 0.154 (0.019, 0.290), respectively]. We did not identify associations between neighborhood safety and adiposity among men and postmenopausal women.

**Conclusions:**

Our data suggest that abdominal adipose tissue distribution patterns are associated with perceived neighborhood safety in some groups, and that patterns may differ by sex and menopause status, with most associations observed among pre-menopausal women. Further research is needed to elucidate whether there are causal mechanisms underlying sex and menopause-status differences that may mediate associations between perceived safety and abdominal adiposity and potential protective factors that may modify this risk.

## Introduction

Adiposity that is distributed centrally in visceral and subcutaneous abdominal depots are thought to increase risks for atherosclerotic heart disease. [1] While both visceral and subcutaneous adipose tissue are associated with cardiometabolic risks in African Americans, visceral fat is a stronger correlate of cardiometabolic risks. [2], [3] African Americans, particularly African-American adult women, are also disproportionally affected by prevalent general and abdominal obesity. [4], [5] Among the many contributors to abdominal adiposity, psychological stress associated with abnormal (blunted and hyper-exaggerated) hypothalamic-pituitary-adrenal (HPA) axis hormone responses is thought to increase abdominal fat storage [6], [7].

Neighborhood social exposures are increasingly thought to contribute to obesity and overweight. [8] Early evidence suggests the lack of perceived neighborhood safety due to crime may contribute to psychological stress and obesity among exposed residents. [9], [10], [11], [12] Stress associated with lack of perceived neighborhood safety is thought to relate to obesity directly via HPA axis disruption, or, via the behavioral and cognitive motivational pathways that may contribute to obesity and overweight. [13], [14] Exposures to unsafe neighborhoods are highly prevalent in African-American communities. [15] However, it is unknown whether perceived neighborhood safety is associated with stress-related abdominal fat patterns, namely, increased visceral abdominal adiposity among African Americans.

We have shown that African-American women who perceived their neighborhoods as unsafe had an increased odds of an elevated waist circumference compared to women who perceived their neighborhoods as safe. This association was observed even after adjustment for potential mediators and confounders, including physical activity and socioeconomic status. [16] Among men, perceived neighborhood safety was not associated with elevated waist circumference.

Our current study examines cross-sectional associations between perceived neighborhood safety, abdominal fat distribution, health behaviors, and socioeconomic factors among African-American men and women in the Jackson Heart Study (JHS). Our analysis is novel in its usage of computed tomography (CT) scan imaging data to examine perceived neighborhood safety in relation to total abdominal fat, and visceral and subcutaneous stores. It is known that patterns of abdominal fat distribution are heavily influenced by sex and menopause status. [17] Our objective is to investigate whether perceived neighborhood safety is associated with abdominal fat distribution (visceral, subcutaneous and total abdominal fat) and anthropometric measures of obesity (body mass index and waist circumference) independent of health behaviors (physical activity, dietary intake and smoking), and socioeconomic factors (income and education). We also examine heterogeneities in these associations by sex and menopause status.

## Methods

### Study design and population sample

The JHS is an observational cohort study of African Americans residing in the Jackson, Mississippi metropolitan area (MSA: Hinds, Madison, and Ranking counties). The objective of the JHS is to identify factors associated with the development of cardiovascular disease in African Americans.

Study design and recruitment methods have been described previously. [18] Participants were recruited from four different sources: 1) 31% (n = 1626) of the JHS participants were recruited from participants of the Atherosclerosis Risk in Communities Study; 2) 17% (n = 921) of the JHS participants were recruited randomly from a commercially available list (AccuData Integrated Marketing, Fort Myers, FL) of all residents 35–84 years in the Jackson MSA; 3) 30% (n = 1569) of the JHS participants were volunteers aged 35–84 years, who were selected to be representative of the overall African-American population in Jackson MSA in terms of age, sex, and socioeconomic characteristics; and 4) the remaining 22% (n = 1185) of the JHS cohort comprised family members. Enumerated families had at least two siblings and four other first-degree relatives who were ≥21 years old and living in the tri-county area. The JHS study protocol was approved by the Institutional Review Boards of Jackson State University, Tougaloo College and the University of Mississippi Medical Center, and all participants provided written informed consent. These analyses were approved by the Partners HealthCare Institutional Review Board.

Between 2000 and 2004, 5,301 African-American men and women completed the first clinical exam. Between 2007 and 2009, 4,203 individuals completed the second clinical exam, of which, 2,884 individuals underwent abdominal CT scanning. After excluding three individuals with unreadable visceral, subcutaneous or total fat images our final analysis cohort was 2,881.

### Measures

#### Outcomes: Computed Tomography

Total abdominal, subcutaneous and visceral fat volumes were assessed via CT scan during the second clinical exam. CT scan participants were limited to non-pregnant individuals weighing less than 350 lbs. Volume (cm^3^) of visceral, subcutaneous, and total abdominal fat were calculated from lower abdomen CT scans between L3-S1, centered at L5, by using previously described procedures [2], [19].

#### Outcomes: Anthropometric measurements

Body mass index (BMI) and waist circumference were measured during the second clinical exam. BMI was calculated from each participant’s height and weight in kg/m^2^. Waist circumference was measured in inches, rounded to the nearest half inch, and converted to centimeters for analysis.

#### Predictor variable: perceived neighborhood safety

Perceived neighborhood safety was assessed from a questionnaire containing an introductory script that described a participant’s neighborhood: “Now I would like to ask you some questions about what it is like to live in your neighborhood. Things about people’s neighborhoods may be important to their health. By neighborhood, I mean the area around where you live. It may include places you shop, religious or public institutions, or a local business district. It is the general area around your house where you might perform routine tasks, such as shopping, going to the park, or visiting with neighbors.” Perceived neighborhood safety was then classified from a single item statement: “This neighborhood is safe from crime.” Individual participants rated their agreement with this statement and responses included: strongly agree, agree, disagree or strongly disagree. Data on perceived neighborhood safety were collected during the third annual follow-up call from the first clinic exam date.

#### Menopause status

Self-reported post-menopausal status was defined based on the questions: “Have you reached menopause?” (N = 1,288 women responded “Yes”) and “Have you had a menstrual period during the past 2 years?” (N = 78 women responded “No”). Women who responded “No” to both questions and were 65 years and older were classified as post-menopausal (N = 3) [20].

#### Covariates

The following covariates were measured during the baseline clinical exam and survey. We used each participant’s self-reported age and sex. Physical activity was measured via an active living index, which was comprised of the frequency and duration of physical activities minus the frequency and duration of sedentary behavior and validated against accelerometers. [21], [22] Individuals were classified as never, former or current smokers. Education was classified into less than high school education, high school graduate/GED, some college or college graduate or higher. Annual family income was scaled for family size. [23], [24] Dietary intake was assessed by a validated food frequency questionnaire adapted for adults living in the Mississippi Delta Region. [25], [26] We assessed participants’ percentages of daily calories consumed from alcohol and protein, and we identified participants who met United States Department of Agriculture recommendations on the proportion of macronutrient intake from carbohydrates and fats [27].

### Statistical analysis

#### Descriptive data

First, we present descriptive median (inter-quartile range) and percentages of demographic, behavioral, and clinical characteristics of participants by sex and menopause status. Next, we present median adipose tissue volume and median anthropometric measurements by categories of perceived neighborhood safety, stratified by sex and menopause status. We performed two-tailed test of statistical significance using the Kruskal-Wallis analysis of variance test to determine the p-for-trend within each measure of adiposity by neighborhood safety levels.

#### Multivariable models

To account for potential effect modification by sex and menopause status we also stratified our multivariable models by sex (male/female) and menopause status (pre and post-menopause). To reduce bias from list-wise deletion of missing covariates we used multiple imputation for missing data using the Markov Chain Monte Carlo method in PROC MI and PROC MIANALYZE in SAS. [28], [29] We converted all categorical covariates to binary dummy variables and imputed all missing data using the independent and dependant covariates in the multiple imputation models. All implausible values were rounded and recoded prior to the PROC MIANALYZE step. To estimate associations between measures of abdominal adiposity, perceived neighborhood safety, and individual-level covariates, we estimated standardized beta coefficients and 95% confidence intervals (CIs) from multivariable regression models using the PROC GLM procedure in SAS. We performed sequential models for men, women (all) and pre- and postmenopausal women (separately) for each measure of adiposity adjusting for perceived neighborhood safety, age, smoking, physical activity, education, income, alcohol, fat, protein and carbohydrate intake. We tested formal interaction terms to assess for sex by perceived safety interactions, and in the models for women, we also separately tested for a statistical interaction between menopause status and perceived neighborhood safety. All tests were two-sided and statistical significance was set at *p*<0.05. All descriptive statistics and multivariable models were analyzed in SAS version 9.3 (SAS Institute, Cary, NC).

#### Sensitivity analysis

A sensitivity analysis using the Fisher’s exact test was performed on individuals weighing more than 350 lbs (n = 39) who were excluded from our analysis to determine whether these individuals differed from the analysis sample. Additionally, we conducted a sensitivity analysis to exclude women missing menopausal status information (n = 14) to determine their influence on the associations we report.

## Results

### Abdominal fat distribution, BMI, and waist circumference measures by sex and menopause status

Selected clinical, demographic, and behavioral characteristics and perceived neighborhood safety measures, stratified by sex and menopause status, are presented in Table 1. Men had a higher visceral fat volume and lower subcutaneous fat volume compared to women (visceral: 842 cm^3^ vs. 742 cm^3^, *p*<0.0001, subcutaneous: 1589 cm^3^ vs. 2555 cm^3^, *p*<0.0001). Compared to premenopausal women, postmenopausal women had higher visceral fat volume but lower subcutaneous fat volume (visceral: 776 cm^3^ vs. 668 cm^3^, *p*<0.0001; subcutaneous: 2513 cm^3^ vs. 2683 cm^3^, *p*<0.01). Men had lower BMI, but higher waist circumference compared to women (BMI: 29 kg/m^2^ vs. 32 kg/m^2^, *p*<0.0001; waist circumference: 102 cm vs. 99 cm, *p*<0.001). Pre- and postmenopausal women had similar BMI and waist circumference sizes (BMI: 33 vs. 32 kg/m^2^, *p*<0.05; waist circumference: 99 vs. 99 cm, *p* = 0.06).

**Table 1:** Selected Demographic Characteristics among Men, Women and Pre- and Postmenopausal Women.

	Males	Females	Females Premenopausal	Females Postmenopausal
	N = 1014	N = 1867	N = 484	N = 1369
**Age, median (IQR), years**	57 (50–67)	60 (51–68)	48 (44–53)	64 (57–71)
**CT imaging, median (IQR), cm^3^**				
Visceral adipose tissue	842 (581–1120)	742 (544–1010)	668 (485–898)	776 (575–1044)
Subcutaneous adipose tissue	1589 (1181–2116)	2555 (1944–3329)	2683 (2002–3514)	2513 (1908–3283)
Total adipose tissue	2548 (1940–3303)	3449 (2659–4377)	3457 (2604–4466)	3439 (2686–4345)
**Anthropometric, median (IQR)**				
Body mass index, kg/m^2^	29 (26–33)	32 (28–37)	33 (28–38)	32 (27–36)
Waist circumference, cm	102 (94–110)	99 (89–109)	99 (89–109)	99 (90–109)
**Behaviors**				
** Smoking, %**				
Never	59.2	75.8	80.8	74.1
Former	26.2	15.7	11.2	17.3
Current	14.6	8.6	8.0	8.6
** Active living index, median (IQR)**	2.25 (1.50–2.75)	2.00 (1.50–2.75)	2.25 (1.63–2.75)	2.00 (1.25–2.75)
** Meets recommended fat intake, %** a	45.5	47.8	39.5	50.6
** ≥15% of total calories from protein, %**	43.7	39.4	40.0	39.2
** Meets recommended carbohydrate intake, %** b	96.6	93.4	95.3	92.8
** Percent alcohol intake, median (IQR), %** c	0.11 (0.01–2.00)	0.02 (0.01–0.14)	0.03 (0.01–0.24)	0.01 (0.01–0.11)
**Socioeconomic Status**				
** Education, %**				
Less than high school	16.2	12.7	3.1	16.1
High school graduate/GED	17.8	20.2	14.9	22.1
Some college	28.7	28.2	35.7	25.6
College graduate or higher	37.3	38.9	46.3	36.2
** Scaled Family Income, median (IQR), $**	39,906 (19,761–57,637)	25,096 (14,000–42,500)	30,000 (17,715–42,500)	23,053 (14,000–42,500)
** Perceived neighborhood safety, %** d				
Strongly agree	14.2	9.4	9.9	9.1
Agree	50.8	47.3	49.0	46.8
Disagree	24.5	28.3	28.7	28.1
Strongly disagree	10.5	15.1	12.4	16.1

a United States Department of Agriculture (USDA) recommendation is ≤35% of total calories from fat.

b USDA recommendation is ≤65% of total calories from carbohydrate.

c Alcohol intake as a percentage of total energy intake.

d Neighborhood safety derived from, “This neighborhood is safe from crime?”.

### Behaviors, socioeconomic characteristics and perceived neighborhood safety by sex and menopause status

Premenopausal women had higher fat intake than postmenopausal women with only 39.5% of premenopausal women meeting recommended fat intake compared to 50.6% of postmenopausal women (*p*<0.0001) (Table 1). Alcohol consumption was relatively low across all groups. Men had lower levels of education attainment, yet, had the highest median scaled family income among all groups. Premenopausal women had higher education than postmenopausal women, but no statistically significant income trends were observed between premenopausal and postmenopausal women. Lack of perceived neighborhood safety was highest among postmenopausal women (16.1%) and lowest among men (10.5%).

### Associations between perceived neighborhood safety, abdominal fat distribution, BMI, and waist circumference

**Figure 1:**
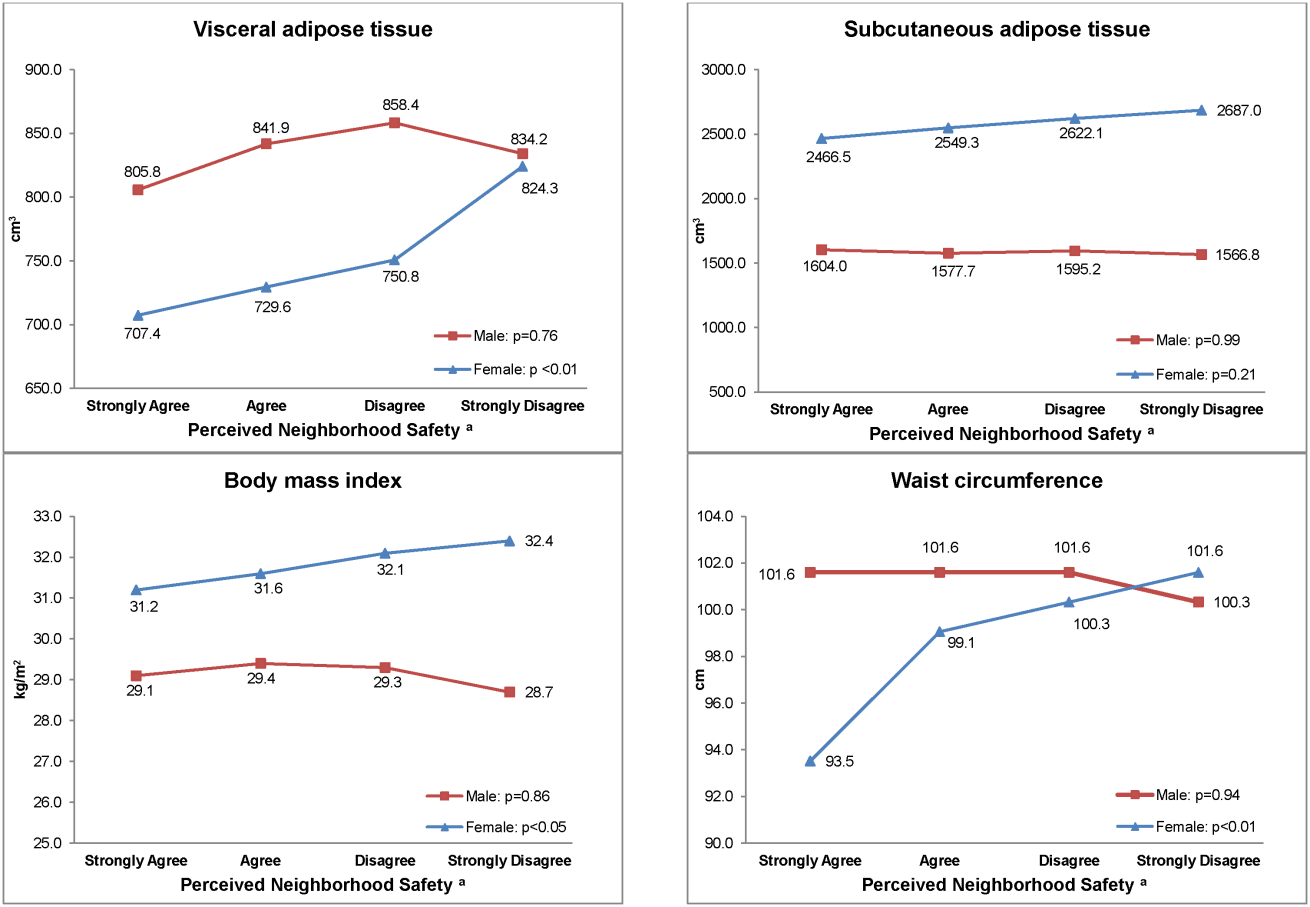
Median Adipose Tissue Volume and Anthropometric Measurements by Perceived Neighborhood Safety by Sex. ^a^Perceived neighborhood safety was derived from the statement “This neighborhood is safe from crime”.

Figure 1 presents abdominal adipose tissue volume and anthropometric measurements by sex and perceived neighborhood safety. We did not observe any association between perceived neighborhood safety, and any of the abdominal fat volumes, or anthropometric measurements among men. In contrast, among women, a linear trend was apparent across all levels of perceived safety for most measures of adiposity. Compared to women who strongly agreed their neighborhood was safe from crime, women who strongly disagreed had significantly higher median visceral fat volume (824.3 cm^3^ vs. 707.4 cm^3^), BMI (32.4 kg/m^2^ vs. 31.2 kg/m^2^) and waist circumference (101.6 cm vs. 93.5 cm). No association was observed between subcutaneous fat volume and perceived neighborhood safety among all women. In contrast to men, women who strongly agreed their neighborhood was safe from crime had significantly lower median total abdominal fat compared to women who strongly disagreed (data not shown).

**Figure 2:**
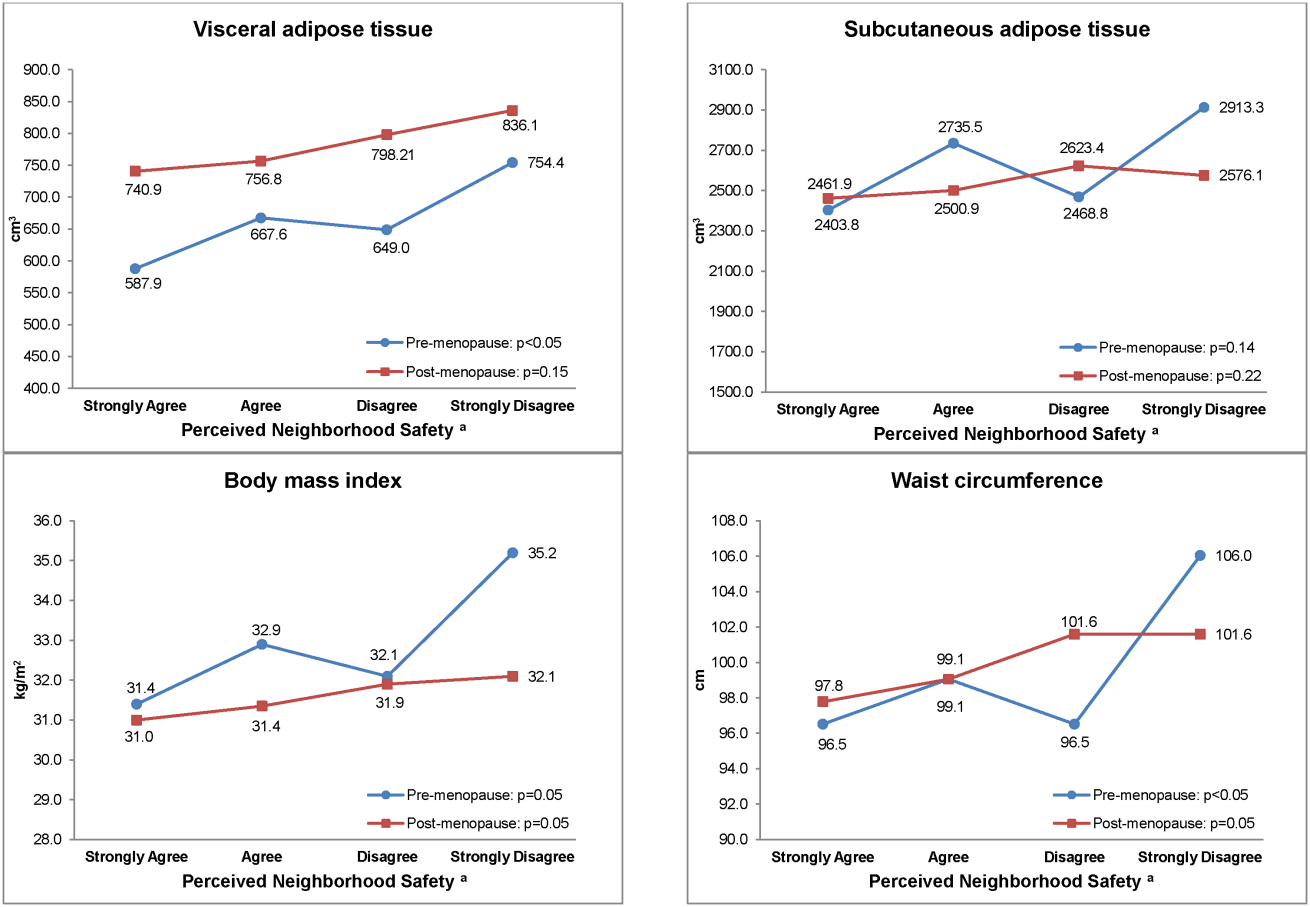
Median Adipose Tissue Volume and Anthropometric Measurements by Perceived Neighborhood Safety by Menopausal Status. ^a^Perceived neighborhood safety was derived from the statement “This neighborhood is safe from crime”.

Figure 2 shows unadjusted associations between perceived neighborhood safety, abdominal adipose tissue volume and anthropometric measurements by menopause status. Premenopausal women who strongly agreed their neighborhood was safe from crime had lower median visceral fat volume (587.9 cm^3^ vs. 754.4 cm^3^) and waist circumference (96.5 cm vs. 106.0 cm) compared to those who felt the least safe. We did not observe any association between subcutaneous fat volume and perceived neighborhood safety among premenopausal women. We also did not observe any association between perceived neighborhood safety and total abdominal fat among both pre- and postmenopausal women (data not shown). Among postmenopausal women, no measures of adiposity were found to be associated with perceived neighborhood safety.

Table 2 presents the regression estimates (95% CIs) from multivariable regression models for men and women for associations between perceived neighborhood safety and abdominal adipose tissue volumes, BMI or waist circumference, adjusted for covariates. Among men, perceived neighborhood safety was not associated with any measure of abdominal tissue adiposity, BMI or waist circumference. In contrast, women who strongly disagreed their neighborhood was safe from crime had significantly higher subcutaneous [Std B 0.073, 95% CI 0.002, 0.145], and total abdominal fat [Std B 0.086, 95% CI 0.011, 0.161], BMI [Std B 0.101, 95% CI 0.028, 0.174] and waist circumference [Std B 0.097, 95% CI 0.021, 0.173] after adjusting for age. When fully adjusting for all covariates, including physical activity, diet, and socioeconomic status, the associations between adiposity and perceived neighborhood safety appeared to be attenuated among all women with exception of BMI [Std B 0.083, 95% CI 0.010, 0.156].

**Table 2:** Standardized Beta Coefficients for Adipose Tissue and Anthropometric Measurements among Men and Women.

	Men N = 1014				Women N = 1867			
	Age Adjusted	95% ConfidenceInterval	Fully Adjusted^a^	95% ConfidenceInterval	Age Adjusted	95% ConfidenceInterval	Fully Adjusted^a^	95% ConfidenceInterval
**Visceral Adipose Tissue**								
Neighborhood Safety								
Strongly agree	Ref.		Ref.		Ref.		Ref.	
Agree	0.004	(−0.097, 0.104)	−0.009	(−0.110, 0.093)	0.049	(−0.048, 0.146)	0.052	(−0.044, 0.147)
Disagree	0.005	(−0.094, 0.104)	0.001	(−0.098, 0.100)	0.065	(−0.027, 0.158)	0.048	(−0.044, 0.140)
Strongly disagree	0.016	(−0.071, 0.104)	0.014	(−0.074, 0.102)	0.075	(−0.005, 0.155)	0.058	(−0.021, 0.136)
**Subcutaneous Adipose Tissue**								
Neighborhood Safety								
Strongly agree	Ref.		Ref.		Ref.		Ref.	
Agree	−0.011	(−0.108, 0.085)	−0.007	(−0.103, 0.088)	0.027	(−0.056, 0.110)	0.028	(−0.054, 0.110)
Disagree	0.003	(−0.092, 0.098)	−0.001	(−0.094, 0.094)	0.051	(−0.027, 0.130)	0.038	(−0.040, 0.117)
Strongly disagree	0.012	(−0.073, 0.096)	0.013	(−0.070, 0.096)	0.073*	(0.002, 0.145)	0.060	(−0.010, 0.131)
**Total Adipose Tissue**								
Neighborhood Safety								
Strongly agree	Ref.		Ref.		Ref.		Ref.	
Agree	−0.007	(−0.105, 0.091)	−0.010	(−0.108, 0.089)	0.037	(−0.051, 0.125)	0.039	(−0.046, 0.125)
Disagree	0.004	(−0.093, 0.101)	−0.00002	(−0.097, 0.097)	0.065	(−0.018, 0.148)	0.049	(−0.033, 0.130)
Strongly disagree	0.017	(−0.071, 0.105)	0.018	(−0.070, 0.105)	0.086*	(0.011, 0.161)	0.070	(−0.003, 0.143)
**Body Mass Index**								
Neighborhood Safety								
Strongly agree	Ref.		Ref.		Ref.		Ref.	
Agree	−0.008	(−0.107, 0.092)	−0.008	(−0.108, 0.092)	0.032	(−0.053, 0.118)	0.034	(−0.052, 0.119)
Disagree	−0.004	(−0.101, 0.093)	−0.011	(−0.108, 0.085)	0.067	(−0.013, 0.148)	0.050	(−0.031, 0.131)
Strongly disagree	−0.008	(−0.096, 0.080)	−0.009	(−0.097, 0.079)	0.101*	(0.028, 0.174)	0.083*	(0.010, 0.156)
**Waist Circumference**								
Neighborhood Safety								
Strongly agree	Ref.		Ref.		Ref.		Ref.	
Agree	−0.001	(−0.098, 0.096)	−0.004	(−0.102, 0.093)	0.032	(−0.058, 0.122)	0.034	(−0.057, 0.125)
Disagree	0.011	(−0.084, 0.106)	0.0004	(−0.094, 0.095)	0.081	(−0.002, 0.164)	0.056	(−0.028, 0.140)
Strongly disagree	−0.001	(−0.087, 0.084)	−0.008	(−0.093, 0.076)	0.097*	(0.021, 0.173)	0.073	(−0.002, 0.148)

Notes: Standardized multivariable regression models imputed for missing covariates via multiple imputation in SAS.

a Models adjusted for age, smoking, physical activity, education, income, alcohol, fat, protein and carbohydrate intake.

*p<0.05.

Table 3 presents regression estimates (95% CIs) for associations between perceived neighborhood safety, abdominal adipose tissue volumes, BMI and waist circumference among women by menopause status. After fully adjusting for covariates, premenopausal women who strongly disagreed their neighborhood was safe had significantly higher visceral fat [Std B 0.150 95% CI 0.014, 0.285], total abdominal fat [Std B 0.154, 95% CI 0.019, 0.290], BMI [Std B 0.160, 95% CI 0.021, 0.299], and waist circumference [Std B 0.142, 95% CI 0.003, 0.280] compared to premenopausal women who strongly agreed their neighborhood was safe. For premenopausal women, the association between perceived neighborhood safety and subcutaneous fat was attenuated after fully adjusting for all covariates. Among postmenopausal women, we did not observe an association between perceived neighborhood safety, and visceral fat, subcutaneous fat, total abdominal fat, BMI or waist circumference.

**Table 3:** Standardized Beta Coefficients for Adipose Tissue and Anthropometric Measurements among Pre- and Postmenopausal Women.

	Premenopausal Women N = 484				Postmenopausal Women N = 1369			
	Age Adjusted	95% ConfidenceInterval	Fully Adjusted^a^	95% ConfidenceInterval	Age Adjusted	95% ConfidenceInterval	Fully Adjusted^a^	95% ConfidenceInterval
**Visceral Adipose Tissue**								
Neighborhood Safety								
Strongly agree	Ref.		Ref.		Ref.		Ref.	
Agree	0.072	(−0.091, 0.235)	0.067	(−0.094, 0.228)	0.030	(−0.079, 0.138)	0.033	(−0.072, 0.137)
Disagree	0.067	(−0.086, 0.221)	0.069	(−0.084, 0.221)	0.056	(−0.043, 0.154)	0.035	(−0.061, 0.131)
Strongly disagree	0.174*	(0.040, 0.309)	0.150*	(0.014, 0.285)	0.044	(−0.042, 0.129)	0.028	(−0.056, 0.111)
**Subcutaneous Adipose Tissue**								
Neighborhood Safety								
Strongly agree	Ref.		Ref.		Ref.		Ref.	
Agree	0.107	(−0.055, 0.270)	0.090	(−0.072, 0.252)	−0.012	(−0.121, 0.097)	−0.006	(−0.113, 0.100)
Disagree	0.060	(−0.096, 0.216)	0.047	(−0.109, 0.203)	0.036	(−0.065, 0.136)	0.025	(−0.075, 0.124)
Strongly disagree	0.154*	(0.022, 0.286)	0.128	(−0.008, 0.264)	0.033	(−0.054, 0.120)	0.024	(−0.062, 0.110)
**Total Adipose Tissue**								
Neighborhood Safety								
Strongly agree	Ref.		Ref.		Ref.		Ref.	
Agree	0.109	(−0.051, 0.270)	0.093	(−0.067, 0.253)	−0.001	(−0.114, 0.112)	0.005	(−0.104, 0.113)
Disagree	0.073	(−0.080, 0.227)	0.062	(−0.091, 0.215)	0.048	(−0.056, 0.151)	0.031	(−0.069, 0.132)
Strongly disagree	0.184*	(0.052, 0.316)	0.154*	(0.019, 0.290)	0.042	(−0.046, 0.130)	0.030	(−0.056, 0.116)
**Body Mass Index**								
Neighborhood Safety								
Strongly agree	Ref.		Ref.		Ref.		Ref.	
Agree	0.101	(−0.068, 0.270)	0.087	(−0.082, 0.255)	−0.006	(−0.114, 0.102)	0.001	(−0.102, 0.103)
Disagree	0.066	(−0.092, 0.226)	0.051	(−0.109, 0.211)	0.055	(−0.044, 0.154)	0.040	(−0.055, 0.136)
Strongly disagree	0.198*	(0.063, 0.334)	0.160*	(0.021, 0.299)	0.059	(−0.029, 0.134)	0.046	(−0.040, 0.132)
**Waist Circumference**								
Neighborhood Safety								
Strongly agree	Ref.		Ref.		Ref.		Ref.	
Agree	0.085	(−0.076, 0.245)	0.062	(−0.096, 0.220)	−0.005	(−0.109, 0.098)	0.001	(−0.102, 0.103)
Disagree	0.083	(−0.072, 0.237)	0.063	(−0.089, 0.215)	0.062	(−0.035, 0.158)	0.040	(−0.055, 0.136)
Strongly disagree	0.189*	(0.054, 0.324)	0.142*	(0.003, 0.280)	0.055	(−0.028, 0.138)	0.046	(−0.040, 0.132)

Notes: Standardized multivariable regression models imputed for missing covariates via multiple imputation in SAS.

a Models adjusted for age, smoking, physical activity, education, income, alcohol, fat, protein and carbohydrate intake.

*p<0.05.

### Sensitivity analysis

During exam two, there were 39 individuals over 350 lbs who did not have a CT scan. Of the 39 individuals who did not receive a CT scan, only 35 had information on neighborhood safety. When compared to the study sample (N = 2881), these individuals were slightly younger (p<0.0001), more likely to be male (p<0.001), and women were less likely to have reached menopause (p<0.05). This group had lower family income (p = 0.05) but did not differ in any substantial way by perceived neighborhood safety (p = 0.38), education (p = 0.12) and smoking status (p = 0.06) when compared to the study sample.

We also performed a sensitivity analysis for all females, excluding the 14 women who were missing menopause status (N = 1853). Adjusting for age, and fully adjusting for all covariates, similar trends in associations were observed between perceived safety and all measures of adiposity when compared to the analysis for all females (N = 1867).

## Discussion

Our study found increased abdominal fat deposition associated with a lack of perceived neighborhood safety among African**-**American women in the Jackson Heart Study. Specifically, premenopausal women who perceived their neighborhoods as most unsafe had higher visceral fat, total abdominal fat, and a higher BMI and waist circumference compared to premenopausal women who felt most safe. No independent associations between neighborhood safety and fat patterning were identified among postmenopausal women or men.

Our study adds to the literature by showing a lack of perceived neighborhood safety is associated with increased *abdominal* fat volume among pre-menopausal women. Among pre-menopausal women who felt most unsafe, we observed visceral fat tissue volumes that were as high as the visceral fat tissue volumes characteristic of men and older post-menopausal women in our study. Our study is novel in observing this association in visceral compartments, hypothesized to be associated with psychological stress pathways.

Psychological distress pathways are thought to contribute to visceral abdominal obesity via physiologic responses such as aberrant cortisol regulation, as well as behavioral responses including emotional eating and insufficient exercise. [30], [31], [32] We did not test specific pathways related to measured psychological distress, cortisol regulation, physical activity or differential eating in this study. However, our results are concordant with observations of associations between neighborhood safety and general adiposity among men and women in other cohorts where these pathways were formally tested. In a multi-ethnic sample, Burdette et al. tested whether exposure to “neighborhood disorder,” including the perception of safety due to crime, leads to psychological distress and behavioral responses that mediate obesity risk. The authors demonstrated that the association between neighborhood disorder and BMI was mediated by perceived psychological stress. [31] Further, the association between perceived psychological distress and BMI was fully mediated by poor self-rated diet quality, and partially mediated by self-reported irregular physical exercise. [31] We did find that, collectively, measures of physical activity and diet attenuated the associations we observed, and these factors, particularly physical activity, likely mediated relations between perceived safety and adiposity in our study as well.

Additionally, our results in pre-menopausal women are consistent with findings in the Fragile Families and Child Wellbeing Study, which examined associations among women with young children. In that cohort, a lack of perceived neighborhood safety was associated with a 1 kg/m^2^ increase in BMI, in the lowest neighborhood safety tertile compared to the highest tertile. [33] Prior studies have found associations between BMI and perceived safety, or perceived psychosocial hazards among men and women, though it is not clear whether there were differential effects by sex, or by life stage among women [9], [12].

Our findings did not show statistically significant interactions suggesting differences in associations with perceived neighborhood safety by sex or menopause status, and the results we observed generate the hypotheses that associations may differ by sex and menopause status, but do not present conclusive causal evidence to this effect. It is unclear why the observed associations between perceived neighborhood safety and abdominal adiposity were seen among premenopausal women, but not among men, or postmenopausal women. We were unable to identify additional studies that examined perceived neighborhood safety in relation to measures of central adiposity by sex. However, our findings are biologically plausible and may be partially explained by experimental studies documenting sex differences in associations between HPA-axis stimulation, sex hormone regulation, and patterns of fat accumulation that suggest sex hormone differences in physiological responses to stress. [34] For example, abdominal fat accumulation is known to be promoted partially by high androgen hormone production as well as cortisol stimulation. In an experimental human study, Vicennati et al. simulated HPA-axis activation via pharmacological stress with adrenocorticotropic hormone. [35] Cortisol responses tended to be associated with *lower* androgen indices in obese men, but *excess* androgen among obese premenopausal women, suggesting susceptibility to excess abdominal fat accumulation in premenopausal women under stressful conditions. [35] Alternatively our findings may relate to sex, life stage, or cohort differences in stress perception, specifically, one’s susceptibility to perceived stress stimuli, given one’s past experiences. For example, the North Carolina Pitt County study studied BMI changes in African-American men and women longitudinally over 13 years, and found that higher levels of perceived stress measured at baseline among women were associated with greater percentage increases in BMI. [36] Similar findings were not found among men, suggesting that different thresholds for perceived stress may potentially modify different weight statuses between African-American men and women. [36] In our study, postmenopausal women were most likely to rate their neighborhoods as unsafe. Therefore, differential stress perception susceptibility may not fully explain the results we observed. Instead, cohort, life stage and sex effects may better explain our results.

Our study has important limitations. Due to the non-experimental design of the study, we cannot draw causal inferences from the reported associations. Notably, measurements of menopause status (examined at the baseline clinical exam 1, 2000–2004), perceived safety (examined during the 3^rd^ annual follow-up from clinical exam 1, 2003–2007) and abdominal fat (examined at the clinical exam 2, 2005–2008) were measured during different times. The time between exams may contribute to a reduced effect size and possible attenuation of associations between abdominal fat and perceived safety. Although we accounted for menopause status, there were a few women (n = 3) who reported having menses after the age of 65, which may have contributed minimal misclassification bias in assigning menopause status. These women were reclassified as postmenopausal to reduce the chance of misclassification bias due to potential dysfunctional uterine bleeding due to malignancies or other factors. Additionally, we are limited in information on residential mobility and do not have residential history of perceived safety from participants. Information on neighborhood safety was only assessed at a single point in time and does not account for life course exposure to this stressor; incomplete assessment of lifetime exposure may potentially underestimate the associations between lack of safety and adiposity we observe, if we are unable to fully account for cumulative disadvantages in those who are chronically exposed.

Additionally, with a cross-sectional design, we cannot fully account for reverse causation, or the effect of unmeasured confounders that may provide alternative explanations for our results. For example, we did not assess physical environmental characteristics (the presence of fast food stores, sidewalks), or include the socioeconomic status of neighborhoods (e.g., poverty) as covariates, which have been associated with obesity in other studies. [8], [37] Prior work in the JHS shows only modest associations between measures of adiposity and fast food stores, and only modest associations between measures of neighborhood socioeconomic status and BMI, and waist circumference after considering perceived neighborhood safety. [16], [38] We were able to include individual-level measures of physical activity and diet, which reduces model misspecification bias due to omitting built neighborhood characteristics associated with these behavioral factors. However, future studies should examine other aspects of the built environment, including neighborhood lighting, or walkability, which were unavailable for our study.

Furthermore, we cannot completely exclude residual confounding from self-report of physical activity in our analysis. However, the self-reported active living index used in this study has been shown to discriminate among more and less physically active participants, as validated against pedometer and accelerometer data. Thus, this measure should reduce potential bias due to residual confounding from incomplete assessment of differences in physical activity among participants [21], [22].

As previously noted, we evaluated the association between perceived neighborhood safety and indicators of adiposity within subgroups of sex and menopause status. Formal tests of statistical interaction did not indicate that there were subgroup differences; however, there was likely insufficient power to detect formal statistical interactions in these smaller subgroups, which is a limitation of the study sample size. Thus, our analyses provide qualitative evidence that there are sex and menopause-related differences in the associations between perceived safety and adiposity. These hypotheses should be tested and confirmed in larger cohorts.

The main strengths of our paper are the large cohort of African Americans and the use of CT imaging data. In addition to anthropometric measures, the use of CT imaging data allows differentiation of abdominal fat compartments that may be modified by neighborhood effects. However, within larger cohorts, CT scanning may not be available or as feasible, in which ultrasound may be an alternative method to obtain fat depot measurements. [39], [40] Our results also suggest that BMI and waist circumference may be adequate measures to assess risks from unsafe neighborhoods for premenopausal women.

In summary, we observed associations between perceived neighborhood safety and fat deposition patterns among premenopausal women. Future research should focus on intervention studies to provide causal data on how potential relations between perceived neighborhood safety and central adiposity are mediated, and how these might be prevented in susceptible premenopausal women, to reduce their future risks for cardiometabolic disease. We did not observe any associations between neighborhood safety and fat distribution among men and postmenopausal women. Additional studies are needed to confirm these findings and identify potential remediable pathways that may affect fat distribution in men and postmenopausal women to reduce risks in these groups.
